# An update on autophagy disorders

**DOI:** 10.1002/jimd.12798

**Published:** 2024-10-17

**Authors:** Hormos Salimi Dafsari, Diego Martinelli, Afshin Saffari, Darius Ebrahimi‐Fakhari, Manolis Fanto, Carlo Dionisi‐Vici, Heinz Jungbluth

**Affiliations:** ^1^ Department of Pediatrics and Center for Rare Diseases, Faculty of Medicine and University Hospital Cologne University of Cologne Cologne Germany; ^2^ Max‐Planck‐Institute for Biology of Ageing; Cologne Excellence Cluster on Cellular Stress Responses in Aging Associated Diseases (CECAD) Cologne Germany; ^3^ Division of Metabolic Diseases Bambino Gesù Children's Hospital IRCCS Rome Italy; ^4^ Division of Child Neurology and Inherited Metabolic Diseases Heidelberg University Hospital Heidelberg Germany; ^5^ Department of Neurology and F.M. Kirby Neurobiology Center Boston Children's Hospital, Harvard Medical School Boston Massachusetts USA; ^6^ Department of Basic & Clinical Neurosciences Institute of Psychiatry, Psychology & Neuroscience, King's College London London UK; ^7^ Department of Paediatric Neurology, Neuromuscular Service, Evelina London Children's Hospital Guy's and St Thomas' Hospital NHS Foundation Trust London UK; ^8^ Randall Centre for Cell and Molecular Biophysics, Muscle Signaling Section Faculty of Life Sciences and Medicine (FoLSM), King's College London London UK

**Keywords:** autophagy, cellular trafficking, congenital disorders, neurodegeneration, neurodevelopment

## Abstract

Macroautophagy is a highly conserved cellular pathway for the degradation and recycling of defective cargo including proteins, organelles, and macromolecular complexes. As autophagy is particularly relevant for cellular homeostasis in post‐mitotic tissues, congenital disorders of autophagy, due to monogenic defects in key autophagy genes, share a common “clinical signature” including neurodevelopmental, neurodegenerative, and neuromuscular features, as well as variable abnormalities of the eyes, skin, heart, bones, immune cells, and other organ systems, depending on the expression pattern and the specific function of the defective proteins. Since the clinical and genetic resolution of *EPG5*‐related Vici syndrome, the paradigmatic congenital disorder of autophagy, the widespread use of massively parallel sequencing has resulted in the identification of a growing number of autophagy‐associated disease genes, encoding members of the core autophagy machinery as well as related proteins. Recently identified monogenic disorders linking selective autophagy, vesicular trafficking, and other pathways have further expanded the molecular and phenotypical spectrum of congenital disorders of autophagy as a clinical disease spectrum. Moreover, significant advances in basic research have enhanced the understanding of the underlying pathophysiology as a basis for therapy development. Here, we review (i) autophagy in the context of other intracellular trafficking pathways; (ii) the main congenital disorders of autophagy and their typical clinico‐pathological signatures; and (iii) the recommended primary health surveillance in monogenic disorders of autophagy based on available evidence. We further discuss recently identified molecular mechanisms that inform the current understanding of autophagy in health and disease, as well as perspectives on future therapeutic approaches.

## 1 INTRODUCTION

Inborn errors of metabolism affect cellular pathways with often highly‐conserved molecular functions that can result in a broad disease spectrum, ranging from lethal multisystem disease to (neuro)developmental disorders (NDDs) of variable severity. The last two decades have seen significant advances in the molecular resolution of monogenic metabolic disorders through the widespread introduction of massively parallel sequencing (exome and genome sequencing) into clinical diagnostics. These novel approaches have focused attention on metabolic pathways that have not been previously considered a common cause of disease, despite their fundamental biological importance.

Autophagy, the digestion and recycling of intracellular material through a highly specialized molecular machinery, is a fundamentally important cellular pathway specifically implicated in human disease only relatively recently. Following the first attribution of the autophagy disorder Vici syndrome to biallelic variants in the ectopic P‐granules 5 autophagy tethering factor gene (*EPG5*),^1^, ^2^, ^3^ more than 50 disorders with primary or secondary defects in autophagy have now been described. These conditions share a considerable overlap in molecular mechanisms and clinical features and are now commonly referred to as *congenital disorders of autophagy*.

**FIGURE 1:**
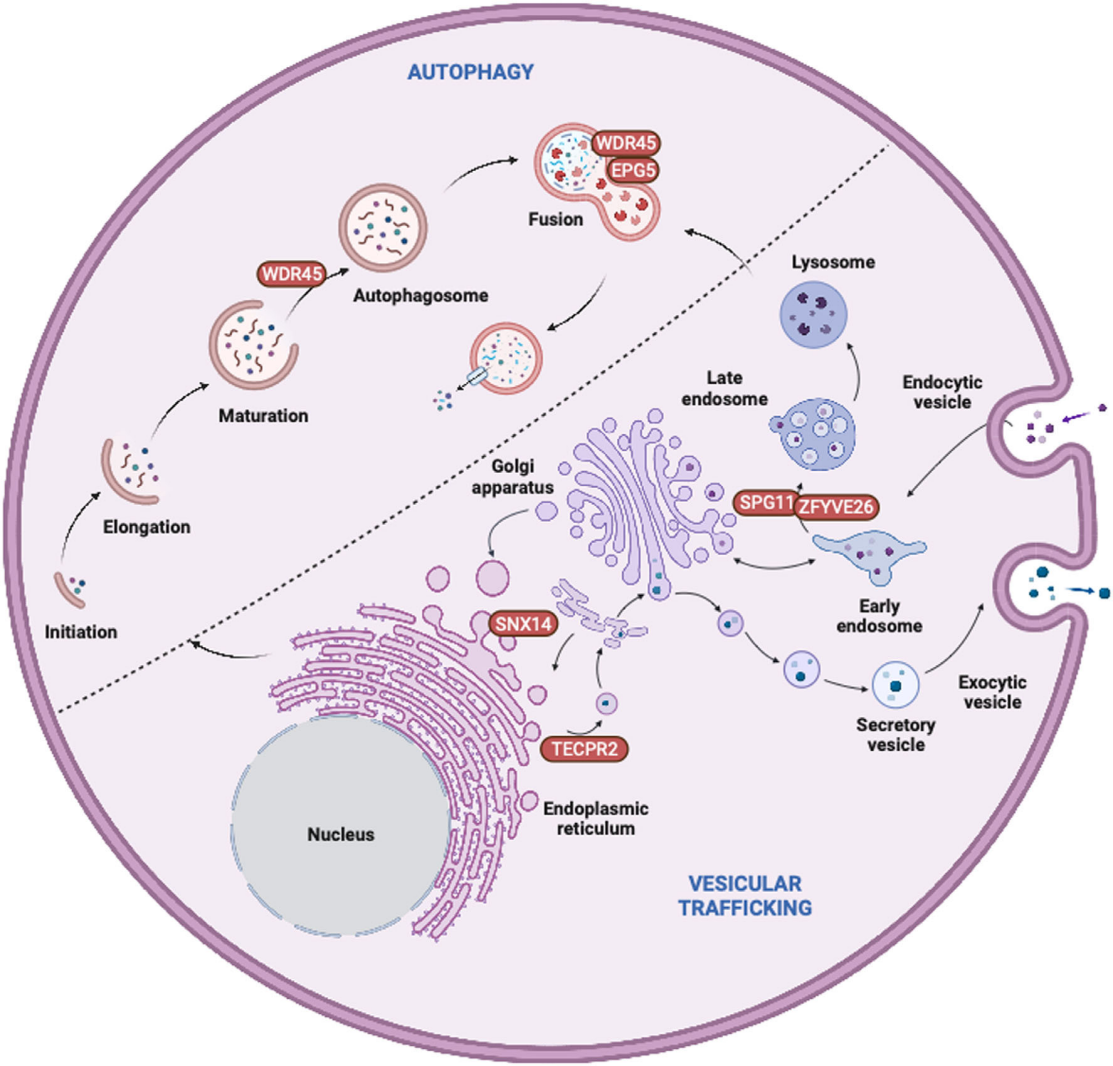
Schematic overview of core autophagy and vesicular trafficking with the genes implicated in the originally described congenital disorders of autophagy highlighted. Upper left: Overview of autophagy from initiation and elongation of a double‐sided membrane to WDR45‐mediated maturation and closure of the autophagosome. Eventually autophagosomes fuse with lysosomes aided by tethering factors such as EPG5 and WDR45, prior to the degradation of defective cargo for recycling through lysosomal acidic hydrolases. Lower right: Overview of endocytosis via vesicles that mature from early to late endosomes/multivesicular bodies mediated by SPG11 and ZFYVE26. Trafficking events between the Golgi apparatus and the endoplasmic reticulum are essential for budding vesicles via mediation by SNX14 for lipid droplets and TECPR2 for autophagosomes. Secretory vesicles may fuse with the plasma membrane for exocytosis. Created with Biorender.com, with permission.

Autophagy exists in different forms: (a) macroautophagy characterized by the delivery of intracellular components through double‐membraned vesicles (or autophagosomes) to the lysosome; (b) microautophagy involving direct substrate engulfment by the lysosomal membrane, and (c) chaperone‐mediated autophagy through recognition of proteins bearing specific motifs by chaperones for delivery and direct importing to lysosomes.^4^ The relevance of macroautophagy (hereafter referred to as autophagy) lies in its fundamental importance for development, cellular survival, and aging. The highly evolutionary conserved autophagy pathway involves several tightly regulated steps: The biogenesis of autophagosomes for engulfing cargo, their eventual fusion with lysosomes for cargo degradation via lysosomal acidic hydrolases and, finally, recycling of digested components and lysosomes within the cytosol (Figure 1).

- *Canonical autophagy and beyond*: Canonical autophagy revolves around the efficient recognition and engulfment of intracellular cargo in a double‐membraned autophagosome that eventually fuses with lysosomes for degradation and recycling.^5^, ^6^ Any dysregulation of autophagy may be due to defects in canonical core autophagy genes (ATGs) or non‐canonical intracellular trafficking genes that regulate the transport of autophagosomes and/or lysosomes,^7^ as well as their interactions through dedicated membrane or cargo contact sites (Figure 1)^8^; in particular the genes implicated in the latter processes may cause simultaneous disruption of autophagy and other closely related intracellular trafficking pathways, resulting in often complex clinical presentations.
- *Cargo selectivity*: Autophagy can occur either in bulk, mainly in response to starvation, or more selectively, targeting, for example, infectious agents (“xenophagy”), defective organelles, in particular mitochondria (“mitophagy”), nuclear lamina (“nucleophagy”), endoplasmic reticulum (“ER‐phagy”), and others. Any dysregulation in selective autophagy may mimic molecular and clinical signs of primary defects in these structures, as demonstrated for mitophagy disorders that may resemble classic *mtDNA*‐related mitochondriopathies.^9^
- *Health in post‐mitotic tissue*: While autophagy is ubiquitous, it is particularly relevant in the homeostatic regulation of post‐mitotic tissues such as the nervous system, heart, bones, and immune cells; any combination of features prominently affecting such tissues should raise the possibility of a disorder affecting intracellular degradation including autophago‐lysosomal disorders. Along similar lines, recent findings in milder autophagy disorders with phenotypes limited to specific organ systems have stimulated the investigation of tissue‐specific pathomechanisms in autophagy disorders, for example, *ATG7*‐related NDDs or *ATG5*‐related ataxia.^10^, ^11^
- *Degradation of toxic substances*: Due to its specificity for macromolecular complexes, defective proteins with an estimated size of over 50 amino acids are degraded via autophagy rather than the ubiquitin‐proteasome system (UPS), the other essential intracellular degradative system. Any deficiency in autophagy may thus trigger the accumulation of toxic substances and prompt cell death, suggesting intersections between autophagic and apoptotic or non‐apoptotic pathways; such an interaction has been recently reported, for examples, with defects in nucleophagy that may lead to karyoptosis,^12^, ^13^, ^14^ or with *WDR45*‐related disorders that may lead to iron accumulation and ferroptosis due to ferritinophagy abnormalities.^15^
- *Expanding phenotypic spectrum of autophagy‐related disorders*: While a complete ablation in core autophagy genes may correspond with developmental defects in eukaryotic organisms, milder disorders associated with defects in autophagy genes are increasingly recognized, often with disease onset only in adolescence or even adulthood. As aging mammals are generally subjected to a physiological decline in autophagy, it is tempting to speculate how genetic defects in autophagy may act synergistically with the normal aging process in “sporadic” neurodegenerative disorders, including amyotrophic lateral sclerosis (ALS), dementia, and Parkinson's disease (PD).

In the following review, we will (i) provide an update on the close links between autophagy and other relevant intracellular pathways; (ii) give an overview of the currently recognized autophagy disorders; (iii) summarize current recommendations for the health surveillance of patients affected by these conditions; and (iv) outline therapeutic perspectives.

## 2 AUTOPHAGY AND INTRICATELY LINKED PATHWAYS IN HEALTH AND DISEASE

Autophagy is a highly conserved cellular process that is unique in its recognition of dysfunctional cargo patterns during autophagosome engulfment, targeting proteins, organelles, and macromolecular complexes. As such, autophagy is expected to be both (i) selective in cargo recognition and (ii) to reliably distinguish defective from healthy cargo. After nucleation, biogenesis and closure of autophagosomes, the subsequent processes include tethering and fusion with lysosomes, and reformation of autophagosomes after lysosomal clearance.^16^

### 2.1 Autophagy and mitochondrial metabolism

Mitochondria play a fundamental role in energy production and cellular metabolism during critical periods of life associated with increased metabolic demands, for example, embryonic development and perinatal adaptation. The close link between primary mitochondrial and autophagy disorders is also illustrated on the clinical level by, for example, prominent mitochondrial abnormalities on muscle biopsies from patients with *EPG5*‐related Vici syndrome,^3^ and, vice versa, cardiomyopathies, movement disorders, and other neurological features shared between primary mitochondrial and autophagy disorders. The removal of damaged or dysfunctional mitochondria through mitophagy is essential for mitochondrial health, and any primary autophagy defects are likely to have detrimental downstream effects on mitochondrial biology. Dysregulation of mitochondrial function secondary to disturbed mitophagy, the mitochondria‐selective form of autophagy, has been implicated in the pathogenesis of various diseases, including *PINK1*‐ or *PRKN*‐related Parkinsonism, *OPTN*‐ or *UBQLN2*‐related amyotrophic lateral sclerosis, and *PNPLA8*‐related mitochondrial myopathy with lactic acidosis.^4^ There have been recent advances in identifying the physiological cargo selectivity of mitochondria in mitophagy. In preparation for mitophagy, UBQLN2 is recruited to poly‐ubiquitinated mitochondria with the chaperone HSP70 in a twofold response which involves (i) promoting UPS‐driven degradation of proteins in the outer mitochondrial membrane (OMM) and (ii) triggering the autophagosomal recognition of the inner mitochondrial membrane receptor prohibitin 2 (PHB2).^17^ To increase the affinity of autophagosomes to mitochondria, Parkin directly binds and ubiquitinates PHB2, enhancing its interaction with microtubule‐associated protein 1A/1B‐light chain 3B (LC3B).^18^ Notably, dysregulation of PINK1/Parkin‐mediated mitophagy was recently identified in mitochondrial methylmalonyl‐coenzyme A mutase (MMUT) deficiency with accumulated mitochondria that ultimately lead to cellular damage.^19^ Hyperhomocysteinemia (HHcy) was recently associated with perturbed autophagy and mitochondrial dysfunction due to Hcy‐induced proteotoxicity leading to sustained ER stress and, ultimately, mTOR activation, and cellular damage.^20^

### 2.2 Autophagy and peroxisomal disorders

Peroxisomes are membrane‐bound organelles that are involved in the catabolism of fatty acids, polyamines, and reactive oxygen species. Primary peroxisomal dysfunction in human disorders causes various neurodegenerative diseases including *ABCD1*‐related adrenal leukodystrophy, Zellweger syndrome due to defects in peroxisomal genes from the PEX family, and other multi‐system disorders including *GNPAT*‐related rhizomelic chondrodysplasia punctata.^21^ Peroxisomal function is tightly linked to mitochondrial health as both organelles are indispensable for beta‐oxidation of fatty acids.^22^

The selective autophagy of peroxisomes is termed pexophagy. Common clinical features of primary peroxisomal and autophagy disorders include epilepsy, movement disorders and myopathies, ocular abnormalities such as cataracts, and multi‐system abnormalities.^23^

Recent studies have implicated a common pathway in both mitophagy and pexophagy driven by the protein NIX.^24^ The SKP1/CUL1/F‐box protein ubiquitin ligase complex SCFFBXL4 on the mitochondrial outer membrane was found to mediate the constitutive ubiquitylation and degradation of the mitophagy receptors NIX and BNIP3 to suppress basal mitophagy levels.^25^ The interaction between mitochondrial and peroxisomal functions in autophagy also includes the selectivity of cargo substrates, as evidenced by a recent study suggesting an association of reduced PEX16 in hepatocytes with reductions in mitochondrial size and autophagy flux.^26^ These recent findings have implicated autophagy as an important link between peroxisomal and mitochondrial health that warrants further investigation; it is possible that secondary peroxisomal disturbances may account at least for some of the severe multi‐system manifestations seen in primary autophagy disease.

### 2.3 Autophagy and lipid metabolism

Lipid metabolism is a complex process that involves the synthesis, storage, and degradation of lipids. Monogenic disorders of lipid metabolism include beta oxidation defects such as *ACADM*‐ or *ACADVL*‐associated metabolic disorders. Lipid droplets (LDs) store lipids that can be utilized for energy production via autophagy, in a process termed lipophagy. Specific disturbances of LD movement or tethering have been linked to congenital disorders of autophagy, including *SNX14*‐related spinocerebellar ataxia, *BICD2*‐related spinal muscular atrophy, and several forms of hereditary spastic paraplegia including SPG3A/*ATL1*, SPG4/*SPAST*, SPG11/*KIAA1840*, and SPG20/*SPART*.^27^

Core autophagy genes have been recently found to aid lipophagy with ATG3 lipidating LC3B to LDs during extended starvation.^28^ Furthermore, LD‐lipidated LC3B acts as a tethering factor, connecting phagophores to LDs and thus promoting degradation.^29^ As any selective autophagy pathway, lipophagy requires distinct receptors for LDs, for example, SPART/spartin.^30^ The oxysterol‐binding protein (ORP8) is involved in lipid transfer and located on LDs, and was recently found to serve as another lipophagy receptor that mediates the encapsulation of LDs by autophagosomal membranes through AMPK phosphorylation, thereby enhancing its affinity in direct interaction with phagophore‐anchored LC3/GABARAPs.^31^ As lipophagy is a relatively recently discovered process, the specific mechanisms for the identification of LDs as autophagy substrates remain elusive. The maintenance of sufficient β‐oxidation levels for ATP production may be related to lipophagy in regulating cell death, suggesting that lipophagy may be relevant for age‐dependent diseases.^32^

### 2.4 Autophagy and lysosomal function

Lysosomes are membrane‐bound organelles that are critical for cellular degradation and recycling through acidic ATPases. The term “lysosomal storage disorders” evokes a recognizable combination of clinical features characterized by coarse facial features, cardiomegaly, as well as neuromuscular and neurodegenerative symptoms. Disorders of endolysosomal biogenesis and lysosomal function include primary lysosomal disorders such as *LAMP2*‐related Danon disease, *GBA*‐related Gaucher disease, *LYST*‐related Chediak‐Higashi syndrome, and *GAA*‐related Pompe disease. Secondary lysosomal disorders evolve further upstream and lead to lysosomal accumulation such as in *NPC1*‐related Niemann‐Pick disease, as well as several mucopolysaccharidoses and glycogen storage disorders.^4^ Shared clinical features of lysosomal and autophagy disorders are multi‐system involvement with facial dysmorphism, hypopigmentation due to abnormal melanocytic trafficking, neurological disorders, and skeletal and cardiac abnormalities.

Recent scientific studies on the relationship between autophagic and lysosomal pathways have focused on (i) non‐canonical autophagy involved in cargo removal, and in (ii) lysosomal repopulation and recycling to sustain autophagy function. Any stall in autophagic flux may prompt cellular attempts to overcome disordered autophagic removal by increased generation of lysosomes. There has been significant progress in the understanding of the Ragulator complex‐mediated interaction of lysosomes and the mammalian target of rapamycin pathway (mTOR) as an alternative pathway to increase lysosomal availability. The mTOR complex 1 (mTORC1) serves as the amino acid sensing machinery in response to nutrient cues and requires the presence of a lysosomal tethering protein, RRAGC. Perturbations in RRAGC affected the size of endolysosomal actin, illustrating the interplay between actin and mTORC1 signaling in the endolysosomal system.^33^ Lysosomal damage was recently implicated in stress granule formation and protein translation inhibition while favoring an ATF4‐dependent integrated stress response.^34^ Damaged lysosomes recruit core stress granule proteins NUFIP2 and G3BP1 along with GABARAP/ATG8 as a novel feature of “ATG8ylation,” a process for the conjugation of the ATG8 family of ubiquitin‐like proteins to membrane lipids that is independent of canonical autophagy. NUFIP2 contributes to mTOR inactivation via the Ragulator complex. This combination of ATG8ylation, stress granules, and mTOR responses to lysosomal damage expands the current understanding of lysosomal activity and may provide the basis for future studies on the pathogenesis of autophagic and/or lysosomal disorders.

During autophagic lysosome reformation (ALR), lysosomes are generated via the recycling of autolysosome membranes. Autolysosomes undergo membrane remodeling and scission to generate membrane fragments that mature into functional lysosomes, particularly during prolonged autophagic flux.^35^ A recently identified lysosomal membrane regeneration pathway independent of autophago‐lysosomal biogenesis includes the lysosomal membrane protein LIMP2 as a lysophagy receptor that binds ATG8 and recruits the RAB7 GTPase‐activating protein TBC1D15 to damaged membranes, providing a scaffold to assemble and stabilize the autophagic lysosomal reformation machinery.^36^ These findings may be relevant for lysosomal dysfunction during infections as TBC1D15 can restore the STING pathway in mitofusin 1 deficiency with disordered mitochondrial fusion.^37^

Recently, the GTPase Vps1 was found to interact with Atg9 as a critical regulator of autophagosome formation in yeast.^38^ Endolysosome and autophagosome maturation are regulated by Rab5 and Rab7, and the Rab5‐to‐Rab7 conversion is mediated by a Rab7 activator, Mon1‐Ccz1. Of note, the identification of a novel Rab5‐GAP TBC1D18 gave more insight into endosome maturation through Mon1.^39^ As any defect in autophagosomes may prompt compensatory cellular mechanisms, upregulation of Rab5‐ or Rab7‐related proteins may at least partially rescue cellular viability.

The role of the endolysosomal system in RNautophagy/DNautophagy (RDA), the autophagic process for direct uptake of nucleic acids by lysosomes for degradation, has recently been further elucidated through the observation that lysosomes take up nucleic acids independently of the intralysosomal acidic pH during RDA.^40^ LAMP2, whose deficiency is associated with Danon disease, has three isoforms (LAMP2A, LAMP2B, and LAMP2C), of which one, LAMP2C, has been identified as an RDA receptor. Future studies may increase understanding of the specific role of RDA in congenital disorders of autophagy.^41^, ^42^ It is likely that DNA repair or RNA splicing disorders lead to accumulation of dysfunctional nucleic acids which in turn may trigger a lysosomal stall due to overactivation of RDA. Of note, several DNA repair disorders such as *ERCC6*‐related Cockayne syndrome share similar clinico‐pathological features with congenital disorders of autophagy, suggesting potentially shared pathomechanisms as a topic for future studies.

### 2.5 Autophagy and vesicular trafficking

Vesicular trafficking is a complex process that involves the transport of proteins and lipids between different cellular compartments. While genes primarily linked to vesicular trafficking strictly speaking do not belong to the core autophagy pathway, their roles extend far beyond trafficking and may affect the regulation of autophagosomes and other autophagy‐relevant processes. Vice versa, considering that autophagy is a highly specialized form of vesicular trafficking, many congenital disorders of autophagy also share similarities with primary trafficking disorders. Several membrane‐related, organellar, and vesicular pathways implicated in cellular disorders of trafficking affect the transport of proteins and organelles converging on neuronal autophagy pathways.^8^ Vesicular trafficking proteins may often be simultaneously involved in multiple selective autophagy pathways and serve as critical regulators of autophagosome maturation and tethering. Autophagosome biogenesis involves the recruitment of various vesicular trafficking proteins, including Rab GTPases, SNARE proteins, and ATG9A. Adaptor protein complexes (AP1–AP5) in particular play a critical role in vesicular trafficking by mediating intracellular sorting of transmembrane proteins along biosynthetic, secretory, endocytic, and lysosomal targeting pathways. APs associate with a particular membrane compartment by interacting with docking proteins, resulting in the recruitment of scaffolding proteins that assemble into protein coats.

Disorders of vesicular trafficking include several movement disorders such as AP‐4‐related hereditary spastic paraplegia, *VPS13C*‐ and *RAB39B*‐related Parkinsonism, *VPS16*‐related dystonia, *VPS13D‐*, *COG5*‐, and *TBC1D23*‐related ataxia, and *NUS1*‐ and *GOSR2*‐related myoclonus.^43^, ^44^, ^45^, ^46^, ^47^, ^48^, ^49^ Rab GTPases are also implicated in various disorders including *RAB7*‐related neuropathy, *RAB27A*‐related Griscelli syndrome, and *RAB18*‐Warburg micro syndrome. Other disorders implicate defective interaction with Rabs, for example, through Rab5 in *ALS2*‐related amyotrophic lateral sclerosis,^50^ through Rab11 in *UNC45A*‐ and *UNC45B*‐related disorders,^51^, ^52^ or through Rab18 in *RAB3GAP1*‐ and *RAB3GAP2*‐related Warburg micro syndrome.^4^

### 2.6 Autophagy and glycosylation

Congenital disorders of glycosylation (CDG) are a heterogeneous group of genetic defects affecting the synthesis and attachment of glycoproteins and glycolipid glycan synthesis. Recently, the dysregulation of the V‐ATPase pump assembly in two congenital disorders of glycosylation has been associated with impaired autophagic flux, perturbed lipid homeostasis, and altered mTOR signaling.^53^, ^54^ Patients with *VMA21*‐related disorders showed isolated chronic liver disease, whereas patients with *ATP6AP2*‐related disorders present with neonatal icterus, immunodeficiency, cutis laxa, and mild cognitive deficits. Of note, VMA21 deficiency was previously associated with X‐linked myopathy with excessive autophagy. *VMA21*‐CDG fibroblasts show an activation of p‐ERK, hypothesized to increase lipogenesis.^53^

The close links between congenital disorders of autophagy and disorders due to defects in protein involved in various aspects of cellular aspects are illustrated in Table 1.

**TABLE 1:** Overlap of phenotypic features in autophagy and cellular trafficking disorders (selection).

Condition	Gene	Clinical feature						
		CNS	Cataract	Cardiomyopathy	Myopathy	Neuropathy	Immunodeficiency	Hypopigmentation
Vici syndrome	*EPG5*	+	+	+	+	+	+	+
Marinescu Sjögren syndrome	*SIL1*	+	+	−	+	+^a^	−	−
CCFDN	*CTDP1*	+	+	−	+	+	−	−
Griscelli syndrome 1	*MYO5A*	+	−	−	?	−	−	+
Griscelli syndrome 2	*RAB27A*	+	−	−	?	−	+	+
Griscelli syndrome 3	*MLPH*	−	−	−	?	−	−	+
Elejalde syndrome	*RAB27A*	+	−	−	?	−	−	+
Chediak Higashi syndrome	*LYST*	+	−	−	+	(+)	+	+
Hermansky Pudlak syndrome 2	*AP3B1*	+	−	−	?	+	−	+
Cohen syndrome	*VPS13B*	+	−	(+)	−	−	+	−
Danon disease	*LAMP2*	+	−	+	+	+	−	−
MEDNIK	*AP1S1*	+	(+)	−	−	+	−	−
MEDNIK‐like	*AP1B1*	+	−	−	−	−	−	−
CEDNIK	*SNAP29*	+	+	−	−	+	−	−
Warburg micro syndrome	*RAB3GAP1, RAB3GAP2, RAB18, TBC1D20*	+	+	−	+	+	−	−

## 3 CONGENITAL DISORDERS WITH DEFECTIVE AUTOPHAGY

**FIGURE 2:**
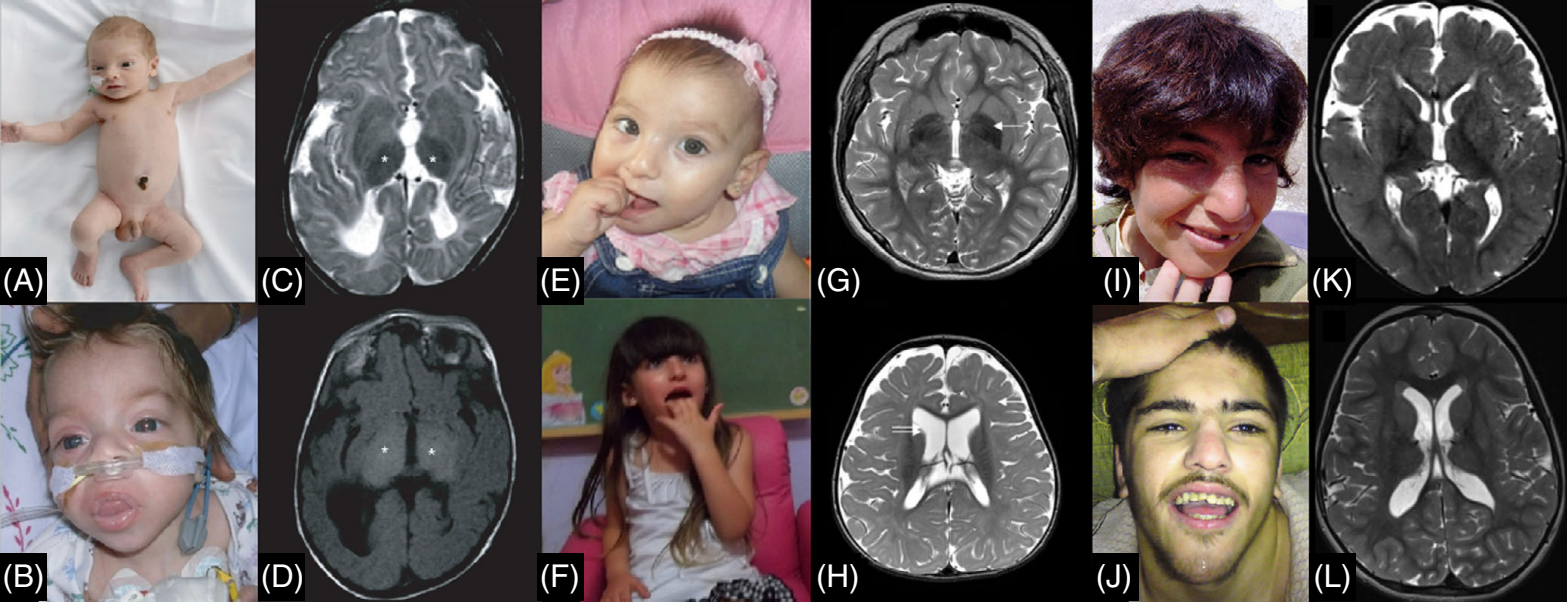
An overview of clinical and radiographic features in selected congenital disorders of autophagy. (A‐D) *EPG5*‐related disorders: Photographs of patients with *EPG5*‐related Vici syndrome with (A) marked hypopigmentation and subtle neurological findings at an early age and (B) coarse facial features suggestive of a storage disorder in a more severely affected patient. (C) Thalamic changes characterized by low signal on T2‐weighted MRI sequences (asterisks) and (D) high signal on T1‐weighted MRI images (D) (asterisks), modified from.^59^ (E–H) *WDR45*‐related disorders: Facial photographs of patients with *WDR45*‐related disorders, modified from.^60^ (G) T2‐hyperintense globus pallidum iron deposition, and (H) diffuse myelin reduction (white arrow) and prominent lateral ventricles (double arrow), modified from.^61^ (I‐L) *AP4S1*‐related disorders: (I,J) Facial photographs of two patients with *AP4S1*‐related spastic paraplegia, modified from.^62^ (K) T2‐hyperintense signal in the forceps minor consistent with the ears of the lynx sign, and (L) short and round T2‐hyperintense signal in the forceps minor, modified from.^63^

Similar to lysosomal storage diseases which often affect multiple organs, congenital disorders of autophagy from the outset were also recognized as having multisystem involvement affecting the central nervous system, eyes, muscle, heart, skin, and immune system. The paradigmatic congenital disorder of autophagy is Vici syndrome, first recognized as a distinct entity in 1988 by Dionisi‐Vici and colleagues and subsequently attributed to biallelic variants in *EPG5* (Figure 2).^1^, ^2^, ^3^, ^55^ Several basic and translational studies focusing on this intriguing condition have provided significant insights into the pathophysiology of the associated NDD, as well as the pertinent multisystem features, in particular immunodeficiency, ophthalmological, and cardiac defects.^3^, ^56^, ^57^, ^58^

In the same year as *EPG5*‐related Vici syndrome, biallelic variants in the WD repeat domain 45 gene (*WDR45*) were identified in patients with beta‐propeller protein‐associated neurodegeneration with brain iron accumulation.^64^ This biphasic disorder presents with childhood‐onset global developmental delay, seizures, and microcephaly, before evolving into a relentlessly progressive disorder characterized by dystonia, Parkinsonism, and cognitive decline in adolescence and adulthood,^15^, ^65^ stimulating further interest into autophagy‐associated disorders and their effects on neurodegeneration.^66^, ^67^ The intricate link between different congenital disorders of autophagy is illustrated by the common role of EPG5 and WDR45 in the autophagosome‐lysosome fusion machinery, and by shared clinical features. WDR45 was initially described to act only on the early steps of autophagosome biogenesis (in particular nucleation) and was only later linked to the final stage of autophagosome‐lysosome fusion.^68^

Based on these two early reports of congenital disorders of autophagy in 2013, previous reviews have described a potential common clinico‐pathological signature of autophagy disorders, combining both multi‐system and primary neurological features, that may aid their diagnosis.^4^, ^69^, ^70^, ^71^ At the time of its introduction in 2016, six conditions (*EPG5*‐related Vici syndrome, *WDR45*‐related BPAN, *SNX14*‐related ataxia as well as *SPG11*‐, *ZFYVE26*‐, and *TECPR2*‐related spastic paraplegia) were included with the concept of congenital disorders of autophagy. However, reflective of recent exponential advances in molecular diagnostics, this novel group of metabolic conditions now encompasses more than 50 conditions, some with primary involvement of key autophagy genes, others with mutations in genes implicated in intricately linked pathways. The overlap of *EPG5*‐related Vici syndrome with other linked conditions was initially recognized through a “reverse search” of *EPG5*‐related features, emphasizing close links between commonality of biological mechanisms and clinical signatures (Table 1).^3^ These disorders may be further sub‐classified by their clinical presentation (Table 2), bearing in mind that despite the predominance of a certain clinical feature there is still considerable clinical overlap.

**TABLE 2:** An overview of congenital disorders of autophagy with core clinical features and key pathogenic mechanisms. Modified from.^4^

Condition	Gene	Role in autophagy and associated pathways
Multi‐system disorders		
Vici syndrome	*EPG5*	RAB7 effector mediating autophagosome‐lysosome fusion
Younis‐Varon syndrome	*FIG4*	Regulates synthesis and turnover of PtdIns(3,5)P_2_
Warburg Micro syndrome	*RAB3GAP1*, *RAB3GAP2*, *RAB18*, *TBC1D20*	Role in autophagosome formation
Chediak‐Higashi syndrome	*LYST*	Lysosomal trafficking
Hermansky‐Pudlak syndrome	*AP3B1*, *AP3D1*	Trafficking from Trans‐Golgi network to endolysosomal system
Griscelli syndrome	*RAB27A*, *MYO5A*	Lysosomal turnover
Neurodegeneration with Brain Iron Accumulation (NBIA)		
Beta propeller‐associated neurodegeneration (BPAN)	*WDR45*/*WIPI4*	Phagophore/autophagosome formation, interaction with EPG5
Cerebellar ataxia		
SCAR20	*SNX14*	Mediates autophagosome‐lysosome fusion and lipid droplet fusion
SCAR25	*ATG5*	Role in autophagic vesicle formation through conjugation to ATG12
Spastic paraplegia		
SPG11	*SPG11*	Autolysosome recycling via ALR
SPG15	*ZFYVE26*	Role in autophagosome formation and autophagosome‐lysosome fusion; autolysosome recycling via ALR
SPG47	*AP4B1*	Trafficking from Trans‐Golgi network to endolysosomal system
SPG49/Hereditary sensory and autonomic neuropathy type IX	*TECPR2*	Putative role in early autophagosome generation by scaffolding at ER exit sites
SPG50	*AP4M1*	Trafficking from Trans‐Golgi network to endolysosomal system
SPG51	*AP4E1*	Trafficking from Trans‐Golgi network to endolysosomal system
SPG52	*AP4S1*	Trafficking from Trans‐Golgi network to endolysosomal system
Amyotrophic lateral sclerosis (ALS)		
FTDALS1	*C9orf72*	Role in autophagosome maturation
ALS2	*ALS2*	Loss‐of‐function causes autophagosome accumulation
FTDALS3	*SQSTM1*	Autophagy receptor required for aggrephagy
ALS4	*SETX*	Role in autophagosome maturation
ALS5	*SPG11*	Autolysosome recycling via ALR
ALS11	*FIG4*	Regulates synthesis and turnover of PtdIns(3,5)P_2_
ALS12	*OPTN*	Selective autophagy receptor in aggrephagy, mitophagy, xenophagy
FTDALS6	*VCP*	Role in autophagy initiation and in mitophagy
FTDALS7	*CHMP2B*	Part of ESCRT‐III complex; role in mitophagy‐specific phagophore closure
Parkinson disease (PD)		
PARK2	*PRKN/PARK2*	Induces mitophagy in concert with PINK1, and within a E3 ubiquitin ligase complex
PARK6	*PINK1*	Induces mitophagy by recruitment of PRKN
PARK20	*SYNJ1*	Autolysosome recycling via ALR
Vacuolar myopathies		
Danon disease	*LAMP2*	Retrograde autophagic abnormalities secondary to impaired autolysosomal fusion
X‐linked myopathy with excessive autophagy (XMEA)	*VMA21*	Retrograde autophagic abnormalities secondary to abnormal lysosomal acidification
Glycogen storage disorders (GSD)		
Glycogen storage disease (GSD) type 2	*GAA*	Secondary to abnormal lysosomal glycogen storage; Retrograde abnormalities of autophagosome formation and autophagosome‐lysosome fusion
Centronuclear myopathies		
X‐linked myotubular myopathy (XLMTM)	*MTM1*	Regulator of PtdIns3P pool; disconnection between fasting and autophagy initiation; failure of ATG machinery to dissociate from PAS site
Neurodevelopmental disorders		
*ATG7*‐related neurodevelomental disorder	*ATG7*	Role in autophagosome maturation

Several genes implicated in congenital disorders of autophagy (Table 2) are associated with widely diverse clinical phenotypes despite often very similar biological roles. For instance, adaptor proteins are generally involved in trafficking events between the Golgi apparatus and endolysosomal system, however, AP‐3‐related disorders present with immunodeficiency and hypopigmentation whereas AP‐4‐related disorders present with progressive spastic paraplegia. Potential explanations may be the tissue‐specific expression of these protein complexes, or remaining gaps in the understanding of their often multiple biological roles.

On the clinical level, at the more severe end of the spectrum, congenital disorders of autophagy have a recognizable combination of clinical features which should prompt the clinician to consider a diagnosis of these conditions (Table 3); however, through the widespread introduction of unbiased massively parallel sequencing a number of milder autophagy‐related disorders have been recognized, reviewed in more detail below.

**TABLE 3:** Common clinico‐pathological features of congenital disorders of autophagy depending on genetic background.

Feature	Genetic background
Disease onset in early childhood/adolescence	Mostly with recessive loss‐of‐function variants core autophagy, selective autophagy and vesicular trafficking genes
Progressive disease course	Mostly with recessive loss‐of‐function variants in core autophagy, selective autophagy and vesicular trafficking genes
Late onset degenerative disease courses	With recessive loss‐of‐function variants in selective autophagy and vesicular trafficking genes
Prominent neurological manifestations	Common to all
Immune system abnormalities	Mostly with genes of late autophagy in interaction with innate immunity
Skin abnormalities	Mostly with genes of autophago‐lysosomal tethering and reformation or lysosomal trafficking (*EPG5*, *LYST*, AP‐3, *RAB27A*, *MYO5A*)
Storage disease phenotype with craniofacial dysmorphia and liver dysfunction	Mostly with genes interacting with lysosomal and peroxisomal pathways
Cataracts	Mostly with recessive loss‐of‐function variants in core autophagy, selective autophagy and vesicular trafficking genes
Cardiomyopathy	Mostly with recessive loss‐of‐function variants in core autophagy and vesicular trafficking genes
Movement disorder	Prominent with recessive variants in core autophagy genes; growing number of monoallelic variants in core autophagy and selective autophagy

While the present review focuses on the relationship of autophagy, wider trafficking and organellar defects, secondary autophagy dysfunction may in principle occur in a much wider range of inborn errors of metabolism, as has been highlighted for glycogen storage disorders and, more recently, primary chromatin disorders.^72^, ^73^ This intriguing overlap between other IEMs and secondarily disturbed autophagy may be the topic of a separate review in future.

### 3.1 Core autophagy disorders

Several disorders have now been associated with pathogenic variants affecting the autophagy core machinery. Primarily neurological disorders include *ATG5*‐related ataxia and *ATG7*‐related NDDs.^10^, ^11^

Biallelic variants in *ATG4D* have been identified in patients with clinical features of a NDD and facial dysmorphism as well as dysfunctional GABARAPL1/ATG8 binding on the cellular level.^74^ ATG4D is involved in mediating both proteolytic activation and delipidation of ATG8 family proteins. Previous publications suggest a similar neurological phenotype in relevant dog models,^75^, ^76^ suggesting a conserved mechanism across species.

Immunodeficiency after infection with Epstein–Barr virus (EBV) was reported in a patient with biallelic variants in *ATG9A*,^77^ encoding a phospholipid scramblase responsible for the translocation of phospholipids between cellular membrane lipid monolayers that mediate autophagosomal membrane expansion. The patient's EBV infection resulted in hyperplastic proliferations of T and B cells in the lung and brain. Peripheral patient blood leukocytes exhibited defects in autophagic activity with defective NFKB signaling.

*De novo* variants in *MYCBP2* were identified in eight patients with a NDD characterized by corpus callosum abnormalities, NDDs, and epilepsy. MYCBP2 interacts with ULK1 in axonal development. Patient variants expressed in the *Caenorhabditis elegans* MYCBP2 orthologue, RPM‐1, led to axonal and behavioral abnormalities including abnormal axonal accumulation of LGG‐1/LC3.^78^

### 3.2 Vesicular trafficking disorders with autophagy abnormalities

A number of additional vesicle trafficking disorders with downstream autophagy abnormalities have been identified recently. Most of these innate errors of intracellular trafficking share clinical features with core autophagy disorders, including both neurological and multisystem features, probably reflecting their downstream dysregulatory effects on autophagy and/or accumulation of autophagy intermediates due to a stall in the core autophagy pathway. Several monogenic disorders may be categorized as both defects in autophagy and vesicular trafficking, for instance *RAB7A*‐related neuropathy or *TECPR2*‐related spastic paraplegia.

Heterozygous *de novo* variants in *RAB5C*, a member of the Rab GTPase family implicated in endocytic protein signaling, were identified in patients with mild to moderate developmental delay and variable features of epilepsy, macrocephaly, and intellectual disability. Studies in *Caenorhabditis elegans* and *Danio rerio* confirmed a damaging effect of variants identified in endocytic pathway function associated with defective development.^79^

Biallelic variants in *VPS33A*, a gene involved in HOPS‐ and CORVET‐related tethering important for autophagosome‐lysosome fusion, were identified in a patient with mucopolysaccharidosis. Fibroblast studies showed a VPS33A decrease due to protein destabilization and proteasomal degradation, increased endolysosomal acidification, and impaired intracellular glycosphingolipid trafficking.^80^ Biallelic variants in *VPS33B* were previously identified in arthrogryposis–renal dysfunction–cholestasis syndrome.^81^

### 3.3 ER‐Golgi trafficking and adaptor protein complex disorders with autophagy abnormalities

Trafficking between the endoplasmic reticulum (ER) and Golgi apparatus requires a number of key regulators to produce vesicles for autophagosome biogenesis. Several proteins may regulate the dichotomous routes from the ER to either the UPS or autophagy, for example, SIL1 implicated in Marinesco‐Sjögren syndrome.^82^

Defects in adaptor protein complex 3 (AP‐3), a protein complex including three AP‐3 subunits, are associated with multisystem and primarily neurological phenotypes. Biallelic variants in *AP3B1* are associated with Hermansky‐Pudlak syndrome type 2 with oculocutaneous albinism, primary immunodeficiency, bleeding diathesis, microcephaly, neurodevelopmental delay, and facial dysmorphism. Biallelic variants in *AP3D1* are associated with a similar spectrum of symptoms. However, biallelic variants in *AP3B2* are associated with early‐onset epileptic encephalopathy,^4^, ^83^ probably reflecting the neuron‐specific expression of AP3B2, in contrast to AP3B1 and AP3D1 that are expressed in a variety of tissues. AP‐4 complex defects were identified with primarily neurological disorders with hereditary spastic paraplegia.^43^, ^44^, ^47^, ^63^, ^84^, ^85^ Similar clinico‐pathological signatures were found in patients with Griscelli syndrome, where biallelic variants in *RAB27A* are associated with a full syndromic picture and biallelic variants in *MYO5A* share NDDs and hypopigmentation but not immunodeficiency.

Biallelic variants in *RABGAP1*, the GTPase‐activating protein of RAB6A for retrograde transport from Golgi to ER, were reported in patients with global developmental delay, microcephaly, bilateral sensorineural hearing loss, seizures, corpus callosum dysgenesis, and facial dysmorphism. Functional analysis of patient cells revealed downregulated mTOR signaling and abnormal localization of early endosomes and lysosomes, while Rabgap1 knockout mice replicated microcephaly and corpus callosum dysgenesis.^86^

Recessive variants in *COG3*, a gene involved in tethering intra‐Golgi transport vesicles, were identified in patients with global developmental delay, severe intellectual disability, microcephaly, epilepsy, facial dysmorphism, and variable neurological findings. Cellular analyses revealed reduced COG3 and COG4 expression and delayed retrograde vesicular recycling.^87^

Biallelic variants in *SEC16B*, a gene involved in ER‐Golgi and ER‐peroxisome trafficking, were found in a patient with osteogenesis imperfecta, vertebral fractures, leg bowing, short stature, and muscular hypotonia. Studies in patient fibroblasts showed accumulated type I procollagen and a trafficking defect with corresponding ER stress, downstream increase of autophagosome formation, and increased apoptosis.^88^

Recessive variants in *TMEM147*, a gene involved in anchoring lamin B receptor (LBR) and with downstream effect on ER homeostasis, were found in patients with coarse facies, developmental delay, intellectual disability, and behavioral problems. Cellular analyses showed accelerated protein degradation via autophagy, LBR mislocalization, and nuclear segmentation.^89^

Biallelic variants in *RINT1*, a key player in ER‐Golgi trafficking, were found in patients with infantile‐onset episodic acute liver failure, spastic paraplegia, ataxia, optic nerve hypoplasia, and facial dysmorphism. Functional and lipidomic analyses in fibroblasts and plasma indicated abnormal lipid‐droplet biogenesis and phosphatidylcholine/phosphatidylserine ratios that may have a likely downstream effect on autophagy.^90^

### 3.4 Defective phosphoinositide signaling with associated autophagy abnormalities

A specific subgroup of proteins involved in the earliest stages of vesicular formation are the phosphoinositides (PIs), a group of membrane phospholipids produced through the local activity of PI kinases and phosphatases that selectively add or remove phosphate groups from the inositol head group. They are critically involved in endolysosomal trafficking and may also have downstream effects on autophagy, as demonstrated in *FIG4*‐related disorders.^91^ Disorders in phosphatidylinositol 4,5 bisphosphate [PI(4,5)P2] phosphatases cause a broad spectrum of NDDs and congenital muscular dystrophy with cataracts and intellectual disability. Heterozygous *de novo* variants in *PIP5K1C* encoding phosphatidylinositol 4‐phosphate 5‐kinase (PIP5KIγ) were found in patients with NDDs, progressive microcephaly, seizures, visual abnormalities, and dysmorphic features. The resulting PI‐signaling dysfunction and an increase in the endosomal pool of PI(4,5)P2 were demonstrated to cause a rise to ectopic recruitment of filamentous actin at early endosomes (EEs) that in turn causes dysfunction in EE trafficking.^92^ In this context it is also of note that congenital disorders of autophagy show remarkable histopathological similarities with the myotubular/centronuclear myopathies due to variants in *DNM2*, *BIN1*, and *MTM1*, the latter in particular encoding a dual specificity phosphatase, myotubularin, with a crucial role in dephosphorylating the key second messenger lipids PI3P and PI3,5P2.^93^ Downstream pathogenic mechanisms in the centronuclear myopathies include secondary autophagy abnormalities due to these defects; other organ involvement suggestive of defective autophagy disorders such cataracts may be part of a more intensive multisystem involvement in these primary neuromuscular conditions.^94^, ^95^

PI4P is generated by four distinct phosphatidylinositol 4‐kinases (PI4K) in mammalian cells. Biallelic variants in *PI4KA* were recently associated with NDD, brain malformations, leukodystrophy, primary immunodeficiency, and inflammatory bowel disease.^96^, ^97^, ^98^ Recently, biallelic variants in *PI4K2A* were identified in patients with developmental and epileptic‐dyskinetic encephalopathy, recurrent infections and death during early childhood.^99^ Neuroimaging showed corpus callosum dysgenesis, diffuse white matter volume loss, and hypoplastic vermis. Cellular studies confirmed the variants' deleterious effect on PI4K2A activity within the endosomal system.

In addition, abnormalities in sphingolipid biosynthesis require trafficking from ER sites to Golgi by the ceramide transfer protein (CERT).^100^ When cells are faced with starvation, PI4K2A and CERT can establish ER and autophagolysosomal contact sites,^101^ Recently, *de novo* missense variants in its gene *CERT1* have been associated with a NDD with epilepsy and craniofacial dysmorphism.^100^

### 3.5 Lipophagy disorders

Abnormalities of lipophagy have been recently recognized as a subgroup of conditions affecting a specialized form of autophagy. Heterozygous variants in *LRP1*, an endocytic receptor gene involved in endocytosis, the phagocytosis of apoptotic cells, were found in patients with developmental dysplasia of the hip (DDH). Heterozygous *Lrp1* knockout mice showed similar phenotypes, while *Lrp1* knockin mice also presented mild DDH phenotypes in heterozygotes and severe phenotypes in homozygotes. On further analyses, *Lrp1* deficiency caused decreased autophagy levels with significant β‐catenin up‐regulation and suppression of chondrocyte marker genes.^102^

### 3.6 Mitophagy disorders

Dominant variants in *VWA8*, a gene encoding a mitochondrial matrix‐targeted protein, were identified in patients with retinitis pigmentosa in a large Chinese family of 24 individuals.^103^ Variants led to reduced *VWA8* expression both at mRNA and protein levels. VWA8 defects led to severe mitochondrial damage, resulting in likely downstream excessive mitophagy and the activation of apoptosis. Knockdown of *VWA8* recapitulated the phenotypes in a *D. rerio* model.

### 3.7 Lysosomal and lysophagy disorders

While the link between autophagy and lysosomal disorders based on the close biological links between the two pathways has been recognized for some time, additional disorders within this spectrum have been recognized recently.

Biallelic variants in *SLC7A14*, a transporter gene for mediating lysosomal uptake of cationic amino acids, were identified in association with loss of inner hair cells and photoreceptors, leading to presynaptic auditory neuropathy and retinitis pigmentosa in mice and humans. Loss‐of‐function variants led to increased basal autophagy and progressive cellular degeneration.^104^

Biallelic variants in *CHMP3*, a core component of the ESCRT‐III complex that is important in the endolysosomal system, were found in five affected individuals with complex hereditary spastic paraplegia. Patient fibroblasts showed significantly reduced levels of CHMP3 and electron microscopy disclosed accumulation of endosomes, autophagosomes, and autolysosomes in patient fibroblasts, which correlated with higher levels of p62 and LC3‐II.^105^

*De novo* missense variants in *RRAGC* were identified in patients with dilated cardiomyopathy, hepatopathy, and brain malformations. Studies of patient fibroblasts revealed increased cell size, as well as dysregulation of mTOR‐related p70S6K and TFEB signaling. Moreover, subcellular localization of mTOR was decoupled from metabolic state.^106^

Mostly *de novo* missense variants in *RRAGD* were identified in eight patients with salt‐losing tubulopathy, hypomagnesemia, and dilated cardiomyopathy. The variants were shown to induce a constitutive activation of mTOR signaling and RRAGD expression along the mammalian nephron including the thick ascending limb and the distal convoluted tubule.^107^

## 4 DIAGNOSIS AND HEALTH SURVEILLANCE

Identifying monogenic disorders of autophagy remains challenging due to the clinical overlap with other conditions. Advances in genetic testing have improved the diagnostic process, complemented by cellular assays for autophagic flux mostly including the markers LC3 and p62/SQSTM1.^6^ Organismic models may provide further insights into in vivo behavioral phenotypes.^108^ The use of molecular genetic, cellular, and organismic approaches should be individualized due to clinical heterogeneity and reflecting the availability of different models. Health surveillance for these disorders typically involves monitoring the phenotypic progression in a multidisciplinary approach. Table 4 summarizes recommended initial investigations and assessments at the point of diagnosis of a suspected congenital disorder of autophagy.

**TABLE 4:** Recommended health surveillance in congenital disorders of autophagy. Modified from.^55^

System/Concern	Evaluation	Comment
Central nervous system involvement	Neurologist	Evaluation of motor, speech, and language development Brain MRI Consider EEG if seizures are a concern Consider specific treatment for movement disorders
Metabolic status	Metabolic pediatrician/physician	Assessment of metabolic status, coordinating care
Musculoskeletal	Orthopedics	Assessment for contractures, scoliosis, and foot deformities
	Physiotherapy and occupational therapy	Assessment for mobility, activities of daily living
Feeding difficulties and failure to thrive	Pediatric gastroenterologist/speech‐language pathologist/dietician	Evaluation of aspiration risk and nutritional status Evaluation for gastric tube placement in case of dysphagia, risk of aspiration, or poor weight gain
Ophthalmologic involvement	Pediatric ophthalmologist	Assesment of visual acuity, refractive error, strabismus, and more complex findings
Sensorineural hearing loss	Audiologist	Evaluation of hearing loss
Cardiac involvement	Pediatric cardiologist	Assessment for cardiac malformations or cardiomyopathy
Immunodeficiency	Pediatric immunologist	Evaluation for symptoms of primary immunodeficiency with specific testing Assesment for thymus a‐/hypoplasia on chest x‐ray
Pulmonary function	Pediatric pulmonologist	Evaluation for aspiration risk and secretion management Consider antibiotic prophylaxis
Hepatic function	Pediatrician; referral to hepatologist as needed	Assessment for liver function
Renal function	Pediatrician; referral to nephrologist as needed	Assessment for kidney function
Ethics consultation	Clinical ethics services	To assess health care decisions in context of best interest of child and values and preferences of family
Genetic counseling	Genetics professionals	Informing affected persons and families regarding nature, inheritance, and clinical course to facilitate medical and personal decision making
Family support and resources	Multi‐disciplinary team including psychologist and social care worker	Assess need for community, social work involvement for parental support, home nursing referral. Referral to palliative care when deemed appropriate by family and health care providers

## 5 MANAGEMENT AND TREATMENT STRATEGIES

Management for congenital disorders of autophagy includes supportive care and symptomatic treatment, aimed at alleviating symptoms, slowing disease progression, and improving quality of life. Disease‐modifying and specific therapies based on individual genetic profiles are currently still at the developmental stage but may lead to more effective and personalized treatments in the future. Collaborative efforts between researchers, clinicians, and patient support organization are crucial for advancing therapy development for these rare disorders. Current approaches include various elements:

1. *Supportive care*: Supportive care measures are essential in managing the symptoms and complications associated with congenital disorders of autophagy (Table 4); those may include physical, occupational, and speech therapy, and nutritional support to address specific needs and improve overall functioning.^55^, ^59^
2. *Symptomatic treatment*: Depending on the specific symptoms and manifestations of the disorder, symptomatic treatments may be initiated to manage seizures, muscle weakness, or movement disorders associated with these disorders (Table 4).
3. *Disease‐modifying therapies*: At present, there are no disease‐modifying therapies. Modulating autophagy through dietary intervention, similar to the use of ketogenic diet in epileptic encephalopathy, may be considered in the future to restore or ameliorate autophagy function and mitigate the underlying disease mechanisms. Another approach being explored is the re‐purposing of existing drugs that have shown potential in modulating autophagy, through screening of drug libraries for compounds with known effects on autophagy. However, with all approaches outlined above, the specific autophagy defect will have to be taken into account, as, for example, general enhancement of autophagic flux may not necessarily alleviate symptoms with blockades further down the pathway.

Currently, more experimental therapeutic strategies may involve stem cell therapy via autologous bone marrow transplant to slow disease progression, for example in immunological diseases such as Chediak‐Higashi syndrome and Hermansky‐Pudlak syndrome.^109^, ^110^ However, the remit of stem cell therapy may be restricted to specific, non‐neuronal tissues, considering the limitations imposed by the blood–brain barrier (BBB). In the future, gene therapy involving various approaches may potentially be leveraged for correcting the underlying genetic defects and restore proper autophagy function.

4 *Future strategies*: The molecular mechanisms underlying monogenic disorders of autophagy are complex and vary depending on the specific disorder. Recent basic research has greatly aided the identification of potential targets for autophagic modulation, particularly in identifying receptors and substrates in selective autophagy and vesicular trafficking. The following considerations have to be taken into account:
  - Failed autophagosome formation and biogenesis: Pathogenic variants in genes encoding ATGs can affect autophagy by impairing the formation or maturation of autophagosomes such as *ATG5*‐ and *ATG7*‐related NDDs and ataxia,^10^, ^11^ and may potentially be amenable to strategies aimed at alleviating core build‐up of autophagic biogenesis including autophagy upregulation or AMPK/mTOR inhibition.
  - Inefficient tethering, trafficking, and reformation of autophagosomes: While the most prominent example is *EPG5*‐related Vici syndrome with deficient autophagolysosomal tethering, a number of other genetic disorders implicate deficient tethering at ER, LDs, organellar recruitment, and efficient reformation of autophagosomes or lysosomes. While upregulation of autophagy via inhibition of the AMPK/mTOR pathway may work for some disorders of early autophagy induction, any modulation of late autophagosome‐lysosome fusion shall be treated with caution to avoid further accumulation of toxic substances.
  - Perturbed interaction with interacting pathways: Recent expansion in genetic disorders of mTOR‐lysosomal and ER‐Golgi complex interactions showed dysregulated autophagy as a common downstream pathway resulting in toxic substance accumulation. While the autophagic disorder may primarily drive cellular degeneration particularly in post‐mitotic tissue, the underlying pathogenesis is not found in core autophagy or related trafficking pathways. The potential of targeting these intricately linked pathways may aid development of novel therapies to rescue both the specific disorder and downstream autophagic dysregulation.

Further research is needed to explore the therapeutic potential of modulating autophagy in the context of monogenic disorders and other related conditions.

## 6 CONCLUSION

Monogenic disorders of autophagy are a group of inherited diseases that affect core and selective autophagy as well as intricately linked pathways, leading to a multitude of clinical manifestations. While the clinical features of monogenic disorders of autophagy are diverse and can affect multiple organ systems, there are common clinico‐pathological signatures that may prompt clinicians to consider this important and evolving group of (neuro)metabolic conditions in their differential diagnosis. Recent basic and translational studies have enabled a deeper understanding of molecular mechanisms underlying these complex and variable disorders and may enable future therapy development.

## AUTHOR CONTRIBUTIONS

HSD wrote the initial manuscript draft. DM, DEF, AS, MF, CDV, and HJ critically revised the manuscript.

## CONFLICT OF INTEREST STATEMENT

The authors have no conflicts of interests.

## ETHICAL APPROVAL

The patient photographs illustrated in this review paper were drawn from previously published original articles with permission from the respective corresponding authors as cited. This review article does not contain any other work on humans or animals.
